# Possible limits and emerging risks of large language models in diagnostic surgical pathology – a dual-specialty perspective

**DOI:** 10.1016/j.clinsp.2026.100967

**Published:** 2026-05-11

**Authors:** Ilker Sengul, Demet Sengul

**Affiliations:** a Thyroidology Unit, Giresun University Faculty of Medicine, Giresun, Turkey; b Division of Endocrine Surgery, Giresun University Faculty of Medicine, Giresun, Turkey; c Department of General Surgery, Giresun University Faculty of Medicine, Turkey; d Department of Pathology, Giresun University Faculty of Medicine, Turkey

**Keywords:** Artificial intelligence, ChatGPT, Surgery, Pathology, Surgical pathology, Thyroidology

Dear Editor,

We read with considerable interest the recent article, “Unveiling the risks of ChatGPT in diagnostic surgical pathology” by Guastafierro et al.^1^ This timely study provides crucial insights into the reliability and potential pitfalls of utilizing ChatGPT, a prominent Large Language Model (LLM) in order to support histopathological diagnosis in thyroidology. We commend the authors for their rigorous methodology, including the crafting of 50 clinico-pathological scenarios across ten subspecialties and evaluation by six expert pathologists.

While the study’s findings highlight key challenges ‒ such as mixed usefulness, variable error rates, and issues with scientific references ‒ we submit that a deeper, pathology-specific critical examination of the possible limits of LLMs is warranted, particularly concerning the rapid technological evolution and clinical integration challenges. Our central thesis is that LLMs are limited for definitive diagnostic tasks due to their unimodal (textual) architecture, yet may serve as assistive tools only if stringent safeguards address emerging, unique risks.

The reliance on the free version of ChatGPT (trained on data up to 2021) raises concerns about the generalizability of the findings, given the rapid advancements that have occurred since the study’s data cutoff. Future LLMs, such as GPT-4o or specialized models utilizing Retrieval-Augmented Generation (RAG) capabilities, may mitigate the observed inaccuracies in scientific referencing and reduce generative errors. However, this improvement introduces a new dependency on the quality and contemporaneity of the source database. Specifically in surgical pathology, where diagnostic criteria evolve rapidly (e.g., frequent updates in WHO breast and thyroid classifications), an LLM retrieving a superseded guideline could be dangerously misleading rather than a simple hallucination, as it carries the veneer of citation-backed authority. This risk is potentially catastrophic, defined as leading to irreversible patient harm (e.g., unnecessary, high-morbidity surgery or failure to diagnose malignancy). Therefore, instead of focusing solely on absolute error rates, the discourse must shift toward defining a diagnosis-specific framework for acceptable AI error in an assistive, rather than replacement, capacity. Defining this ‘acceptable error’ requires risk stratification: an LLM error in suggesting a differential diagnosis for a benign skin lesion may be acceptable for a training pathologist, but the same error in confirming microinvasion in a high-consequence breast carcinoma biopsy is unacceptable. A framework must be fundamentally diagnosis-specific, not based on a blanket error rate.

Guastafierro and colleagues.^1^ correctly identified that scenario variability had the most significant impact on ratings, noting that MC—NR (Multiple-Choice questions without a request for References) was the most influential prompting strategy. This crucial insight requires elaboration. The effectiveness of the MC—NR strategy may stem from its inherent mechanism of constraining the model’s generative scope, thereby reducing the likelihood of catastrophic outputs or “hallucination”. This suggests that prompt engineering serves less as an input optimization technique and more as a safety measure by restricting the scope of AI’s autonomous interpretation.

The conclusion affirming the indispensable human expertise demands careful qualification. While LLMs could potentially support hybrid diagnostic workflows (e.g., triage, quality assurance, or documentation support), the current complexity of integrating visual data, clinical context, and accumulated experience remains fundamentally beyond them. A critical analysis must question the timeframe of this limitation. Is this limitation inherent to AI architecture, or merely a temporary reflection of current computational capabilities? Human expertise encompasses non-computational capabilities like contextual reasoning, ethical judgment, and complex sensorimotor integration that cannot be easily replicated.

To showcase the high-stakes, nuanced role of human synthesis, we draw on our combined expertise in Endocrine Surgery and Pathology. (The previous thyroid example has been removed for refinement, as accepted in R1.) Consider the intraoperative consultation (frozen section) for a pancreatic cyst. The surgeon provides the clinical context: a 2.5 cm cyst in a young female. The pathologist observes mucinous epithelium with moderate atypia. An LLM, queried only with this textual data, might correctly list Intraductal Papillary Mucinous Neoplasm (IPMN) as a possibility. However, the critical distinction between high-risk and low-risk IPMN hinges on integrating multiple data streams: the pathologist must integrate the visual subtleties of papillary architecture, specific immunohistochemical stain results (e.g., MUC1/MUC2 patterns), atypia grading, and the surgeon's real-time description of ductal system involvement. The human expert synthesizes these complex multimodal data points ‒ visual, clinical, and textual ‒ to guide the critical decision between a limited resection and a total pancreatectomy. This multi-factor synthesis process, directly linking pathology findings to irreversible patient-specific surgical risks, is where the LLM fundamentally fails and where clinical judgment remains absolute.

The findings strongly affirm that human oversight and stringent validation protocols are essential. We propose a key research direction: a multi-institutional Delphi study involving pathologists, bioethicists, and legal experts to establish tiered validation criteria for LLMs in diagnostic support. This study must explicitly define how required accuracy thresholds are validated by linking them to the clinical consequence of error (e.g., proposing specific minimum accuracy levels, such as ≥99.5% for high-consequence neuropathology, versus ≥98% for lower-risk skin lesion differential diagnoses). This rigorous, clinically contextualized approach is crucial for establishing a clear regulatory path for AI tools^2^ designed for proper clinical assistance. We thank Guastafierro et al.^1^ for their foundational study of ChatGPT in diagnostic surgical pathology, which provides a critical platform for this necessary discussion.

## Authors’ contributions

Ilker Sengul: Conceptualization; writing-original draft; writing-review & editing.

Demet Sengul: Conceptualization; critical revision on pathology contente; supervision; writing-original draft; writing-review & editing.

## Funding

None declared.

## Data availability

The datasets generated and/or analyzed during the current study are available from the corresponding author upon reasonable request.

## Declaration of competing interest

The authors declare no conflicts of interest.
